# Impact of combined exercise training on the development of cardiometabolic and neuroimmune complications induced by fructose consumption in hypertensive rats

**DOI:** 10.1371/journal.pone.0233785

**Published:** 2020-06-10

**Authors:** Danielle da Silva Dias, Nathalia Bernardes, Filipe Fernandes Stoyell-Conti, Camila Paixão dos Santos, Amanda Aparecida de Araujo, Susana Llesuy, Maria Cláudia Irigoyen, Kátia De Angelis

**Affiliations:** 1 Laboratory of Translational Physiology, Universidade Nove de Julho (UNINOVE), Sao Paulo, Sao Paulo, Brazil; 2 Department of Physiology, Federal University of Sao Paulo (UNIFESP), Sao Paulo, Sao Paulo, Brazil; 3 Department of Surgery, University of Miami, Miami, Florida, United States of America; 4 Instituto Universitario Hospital Italiano, Hospital Italiano de Buenos Aires, Buenos Aires, Argentina; 5 Hypertension Unit, Heart Institute (InCor), School of Medicine, University of Sao Paulo, Sao Paulo, Sao Paulo, Brazil; Max Delbruck Centrum fur Molekulare Medizin Berlin Buch, GERMANY

## Abstract

This study evaluated the impact of combined exercise training on the development of cardiovascular and neuroimmune complications induced by fructose consumption (10% in the drinking water) in hypertensive rats (SHR). After weaning, SHR were divided into 3 groups: SHR (H), SHR+fructose (HF) and SHR+fructose+combined exercise training (treadmill+ladder, 40–60% of maximum capacity) (HFTC). Metabolic, hemodynamic, autonomic, inflammatory and oxidative stress parameters were evaluated in the subgroups (n = 6 group/time) at 7, 15, 30 and 60 days of protocol. Fructose consumption (H vs. HF groups) decreased spontaneous baroreflex sensitivity and total variance of pulse interval at day 7 (7 to 60); increased IL-6 and TNFα in the heart (at day 15, 30 and 60) and NADPH oxidase activity and cardiac lipoperoxidation (LPO) (day 60); increased white adipose tissue weight, reduced insulin sensitivity and increased triglycerides (day 60); induced an additional increase in mean arterial pressure (MAP) (days 30 and 60). Combined exercise training prevented such dysfunctions and sustained increased cardiac IL-10 (day 7) and glutathione redox balance (GSH/GSSG) for the entire protocol. In conclusion, combined exercise training performed simultaneously with exacerbated fructose consumption prevented early cardiovascular autonomic dysfunction, probably trigging positive changes in inflammation and oxidative stress, resulting in a better cardiometabolic profile in rats genetically predisposed to hypertension.

## Introduction

Cardiovascular disease is the leading cause of death worldwide [1]. Moreover, hypertension is the major risk factor for early cardiovascular disease, increasing the risk for range cardiovascular diseases, such as stroke, coronary artery disease, heart failure, atrial fibrillation, and peripheral vascular disease [2].

Indeed, there is a strong association between poor eating habits and cardiovascular disease. Sugar consumption, particularly fructose intake, has been largely studied due to its deleterious effects. Experimentally, high-fructose diets have been shown to lead to moderate hypertension and glucose intolerance, associated with increased levels of plasma insulin, cholesterol and triglycerides [3]. Furthermore, it is well established that fructose overload increases inflammation and oxidative stress markers, which also contribute to increased cardiovascular risk [4]. We have recently shown that in SHR undergoing fructose overload the impairment of baroreflex sensitivity precedes inflammatory and oxidative stress disorders, probably by inducing hemodynamic and metabolic dysfunctions observed in metabolic syndrome [5].

On the other hand, positive effects of exercise training have been demonstrated in the prevention and treatment of hypertension, insulin resistance, diabetes mellitus (DM), dyslipidemia, obesity and metabolic syndrome [6–8]. Indeed, solid evidence has been found for benefits of aerobic exercise training to the cardiovascular and autonomic system, e, g. arterial pressure lowering in hypertensive patients, decreased peripheral vascular resistance, maintenance of left ventricular (LV) mass, increased heart rate variability, reduced systolic arterial pressure variability and improved baroreflex sensitivity. Masson et al. [9] have demonstrated that, regardless of the high pressure levels in SHR, aerobic exercise training promptly restores baroreflex function by disrupting the positive feedback between high oxidative stress and increased pro-inflammatory cytokines secretion within the hypothalamic paraventricular nucleus.

It should be emphasized that resistance exercise training is currently recommended by the American College of Sports Medicine, along with aerobic exercise training (combined exercise training) for individuals with arterial hypertension, peripheral vascular disease, type 2 DM, obesity and other conditions [10]. However, the role of combined exercise training in cardiovascular control, inflammation, and oxidative stress has yet to be fully understood.

Thus, the aim of this study was to evaluate the impact of combined exercise training on the development of cardiovascular and neuroimmune complications induced by fructose consumption in hypertensive rats. We hypothesized that combined exercise training may attenuate the development of autonomic dysfunction in this model, reducing inflammation and oxidative stress, and promoting cardiometabolic improvement.

## Methods

Males spontaneously hypertensive rats (SHR), 30 days old, were obtained from the Animal Facility of the Universidade Nove de Julho. The rats were divided into 3 groups: hypertensive (H, n = 24), hypertensive undergoing fructose overload (HF, n = 24) and hypertensive undergoing fructose overload submitted to the combined exercise training (HFTC, n = 24). Animals from the H group received standard laboratory chow and water ad libitum. Animals from the HF and HFTC groups received fructose in drinking water (D-fructose, 100 g/L) and was initiated at 30 days of life.

The evaluations were performed in 6 rats for each group after 7, 15, 30 and 60 days of fructose or water consumption. All surgical procedures and protocols were approved by the Ethics Committee of Sao Paulo University (Protocol 035/12) and were conducted in accordance with National Institutes of Health -G*uide for the Care and Use of Laboratory Animals*.

### Caloric intake

Chow and water (with or without fructose) consumption were measured weekly. The total caloric intake was calculated using 2.89 kcal per gram of chow consumed and that each ingested gram of fructose corresponds to 4.0 kcal.

### Combined exercise training

Combined exercise training was performed on a motor treadmill (aerobic training) and in a ladder adapted to rats (resistance training), in alternate days.

#### Aerobic exercise training

All animals were adapted to walk and run on a motorized treadmill (10 min/day; 0.3 km/h) before the maximal running test. The aerobic exercise test was performed in sedentary and trained rats as described in detail in a previous study [11]. Aerobic exercise training was performed on a treadmill (Imbramed TK-01, Brazil) at low-to-moderate intensity (40–60% maximal running speed) for 1 h a day, in alternate days with resistance exercise training. To provide a similar environment and manipulation, sedentary animals were placed on the stationary treadmill three times a week.

#### Resistance exercise training

The animals were gradually adapted to the act of climbing before the maximal load test. This is a voluntary exercise protocol, with no aversive (electrical) stimuli to maintain performance, no restraint, and no use of food or water as motivators. The dynamic resistance exercise test was composed of an initial load of 75% of the body weight. After a 2-min resting period, a gradual increase of 15% of body weight was used in the subsequent climbs, as previously described in detail elsewhere. The prescription of resistance exercise training was performed using the normalized value of maximal load for each rat. The resistance exercise training protocol was performed in alternating days with aerobic exercise training at low-moderate intensity with 15 climbs per session and a 1-min time interval between climbs as previously described in details elsewhere [12].

### Blood triglyceride and glucose

In the last day of protocol, before catheterization, triglyceride and blood glucose concentrations were measured (Accucheck and Accutrend, Roche) after 4-hour fasting.

### Cardiovascular and autonomic assessments

Two catheters filled with 0.06 mL saline were implanted in anesthetized rats (80 mg/kg ketamine and 1 mg/kg xylazine) into the carotid artery and jugular vein (PE-10) at day 7, 15, 30 or 60 of protocol (6 animals/group/time) for direct measurements of AP and drug administration, respectively. Rats receiving food and water ad libitum were studied 1 day after catheter placement; they remained conscious and were allowed to move freely during the experiments. An arterial cannula was connected to a transducer (Arterial Pressure XDCR, Kent© Scientific), and AP signals were recorded over a 20-minute period by a microcomputer equipped with an analog-to-digital converter board (Windaq,2-kHz sampling frequency; Dataq Instruments, Inc). The recorded data were analyzed on a beat-to-beat basis to quantify changes in mean arterial pressure (MAP) and heart rate (HR).

For time and frequency domains analysis of cardiovascular autonomic modulation, the time series (three time series of 5 min for each animal) of pulse interval (PI) and systolic arterial pressure (SAP) were cubic spline-interpolated (250 Hz) and cubic spline-decimated to be equally spaced in time after linear trend removal; power spectral density was obtained through the Fast Fourier transformation. Spectral power for low-frequency (LF, 0.20–0.75 Hz) and high-frequency (HF, 0.75–4.0 Hz) bands was calculated by power spectrum density integration within each frequency bandwidth, using a customized routine (Cardioseries). The time-domain variables were: root mean square of the successive differences (RMSSD) and total variance of pulse interval (VAR-PI) for pulse interval (PI); and total variance of systolic arterial pressure (VAR-SAP) for systolic arterial pressure (SAP). The α index in the low-frequency band was calculated only when the magnitude of the squared coherence between the PI and SAP signals exceeded 0.5 (range, 0–1). After coherence calculation, the α index was obtained from the square root of the ratio between PI and SAP variability in the two major low-frequency (LF) band [8]

### Insulin tolerance test

Following hemodynamic evaluation, insulin tolerance test was performed to obtained the constant rate for blood glucose disappearance (KITT) as previous described [13].

### Insulin determination

Insulin concentration was measured in plasma of fasting animals (4 hours) by immunoenzymatic test (ELISA) using a commercial kit (EZRMI-13K / Rat / Mouse insulin, Merck Millipore, USA). Test sensitivity was 0.2 ng/ml. Absorbance was measured at 450 nm in a microplate reader.

### Plasma nitrites

One day after hemodynamic evaluations, the animals were killed and the white adipose tissue, spleen and plasma were immediately collected for analysis.

Nitrites (NO^-^_2_) were determined on plasma using the Griess reagent, in which a chromophore with a strong absorbance at 540 nm is formed by the reaction of nitrite with a mixture of naphthylethylenediamine (0.1%) and sulfanilamide (1%). A standard curve was established with a set of serial dilutions (10^−8^–10^−3^ mol/l) of sodium nitrite.

### Inflammatory mediators in cardiac tissue

IL-6, IL-1β and TNF-α levels in heart was determined using a commercially available ELISA kit (R&D Systems Inc.) in accordance with the manufacturer’s instructions. ELISA was performed in 96-well polystyrene microplates with a specific monoclonal antibody coating. The threshold of sensitivity was 15.0 pg/mL. Absorbance was measured at 540 nm in a microplate reader.

### Oxidative stress profile in cardiac tissue

#### NADPH oxidase

The activity of NADPH oxidase enzyme was determined in homogenate of heart and was evaluated by the production of superoxide determined by plate reader. To perform the assay, 50 mM phosphate buffer containing 2 mM EDTA and 150 mM sucrose, 1.3 mM NADPH and 10 μl of cardiac tissue sample were used. Superoxide production was expressed in μmol/mg protein [14].

#### Superoxide anion

Superoxide anion was determined in the ventricular tissue homogenate by calculating the rate of oxidation of adrenaline at 480 nm. Briefly tissue homogenate was added to glycine buffer followed by addition of catalase (494 μM). Absorbance was set to zero. Adrenaline (60 μM) was added and absorbance was recorded at 480 nm for 2 mins. Amount of superoxide anion was express(15)ed as mmol/mg protein [15].

#### Hydrogen peroxide concentration

The assay was based on the horseradish peroxidase- (HRPO) mediated oxidation of phenol red by H_2_O_2_, leading to the formation of a compound measurable at 610 nm. Cardiac tissue was incubated for 30min at 37°C in 10mmol/L phosphate buffer consisting of 140mmol/L NaCl and 5mmol/L dextrose. Supernatants were transferred to tubes with 0.28mmol/L phenol red and 8.5U/mL HRPO. After 5 min incubation, 1 mol/L NaOH was added and it was read at 610 nm [16].

#### Membrane lipoperoxidation by chemiluminescence

The chemiluminescence (CL) assay was carried out with an LKB Rack Beta liquid scintillation spectrometer 1215 (LKB Producer) in the out-of-coincidence mode at room temperature. Supernatants were diluted in 140 mM KCl and 20 mM sodium phosphate buffer, pH 7.4, and added to glass tubes, which were placed in scintillation vials; 3 mM tert-butyl hydroperoxide was added, and CL was determined up to the maximal level of emission [17].

#### Determination of protein oxidation by carbonyls

*S*amples were incubated with 2,4-dinitrophenylhydrazine (DNPH 10 mM) in a 2.5 M HCl solution for 1 h at room temperature in the dark. Samples were vortexed every 15 min. Subsequently, a 20% trichloroacetic acid (w/v) solution was added and the solution was incubated on ice for 10 min and centrifuged for 5 min at 1000 g to collect protein precipitates. An additional wash was performed with 10% trichloroacetic acid (w/v). The pellet was washed three times with ethanolethyl acetate (1:1) (v/v). The final precipitates were dissolved in 6 M guanidine hydrochloride solution and incubated for 10 min at 37°C, and absorbance was measured at 360 nm [18].

#### Antioxidant enzyme activities

The quantification of SOD activity was based on the inhibition of the reaction between O2˙^−^ and pyrogallol [19]. CAT activity was determined by measuring the decrease in H_2_O_2_ absorbance at 240 nm [20]. GPx activity was expressed as nmol peroxide/hydroperoxide reduced/ min/mg protein and was based on the consumption of NADPH at 480 nm [21].

### Statistical analysis

Data are expressed as mean±SEM. The Levene test was used to evaluate data homogeneity. A two-way analysis of variance followed by the Student-Newman-Keuls test was used to compare groups. Significance level was established at p≤0.05.

### Sources of funding

This study was supported by CNPq (457200/2014-6; 309292/2014-0) and FAPESP (2015/11223-6); CAPES (88881.062178/2014-01). Kátia De Angelis and Maria-Claudia Irigoyen are recipients of CNPq Fellowship (CNPq-BPQ).

## Results

### Tissues weight and metabolic evaluations

**Fig 1:**
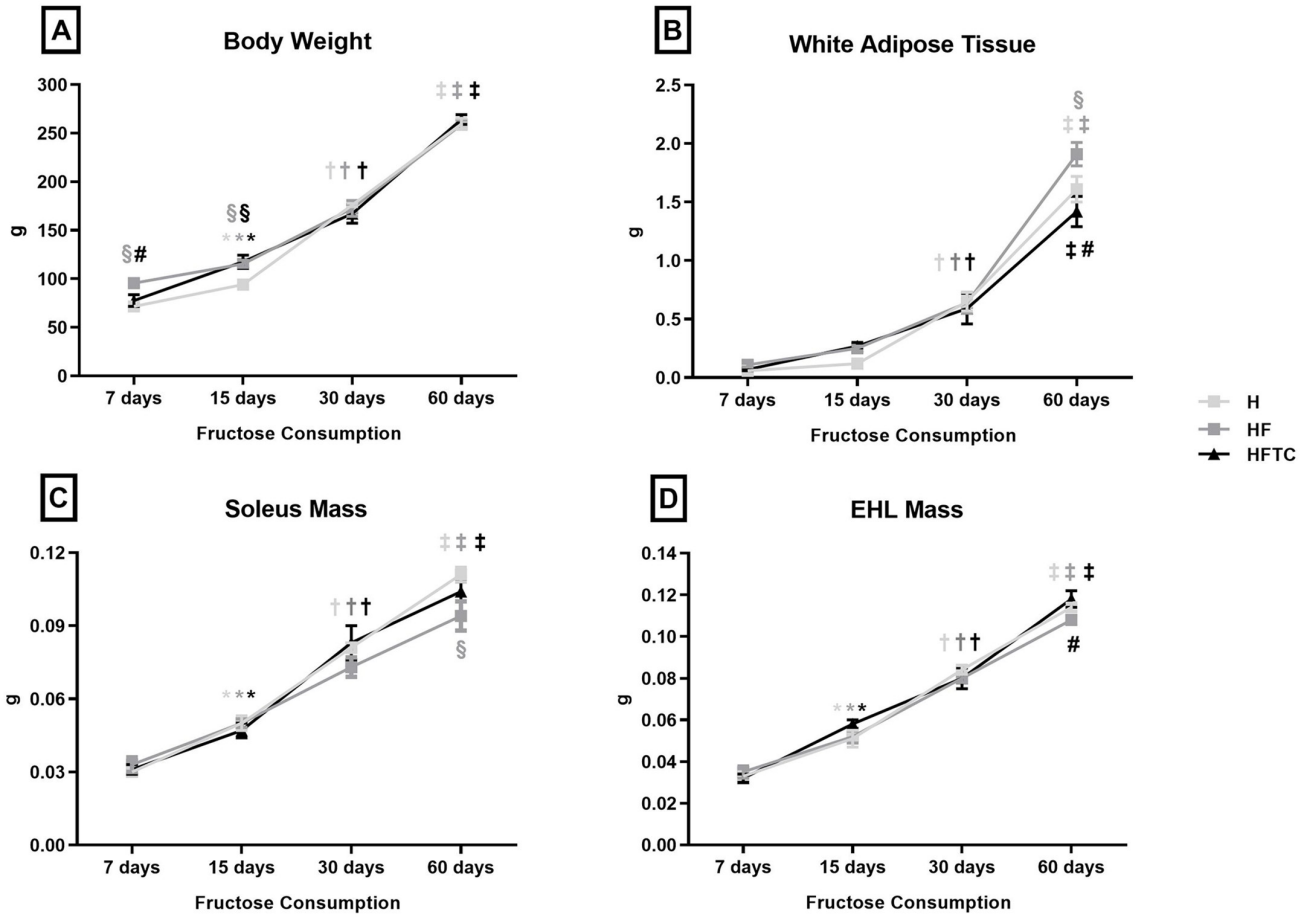
A) body weight, B) white adipose tissue weight, C) soleus mass and D) extensor hallucis longus (EHL) mass of the hypertensive (H), hypertensive + fructose (HF) and hypertensive + fructose + combined physical training (HFTC) groups at 7, 15, 30 and 60 days. * p <0.05 vs. 7 days in the same group; † p <0.05 vs. 7 and 15 days in the same group; ‡ p <0.05 vs. 7, 15 and 30 days in the same group; § p <0.05 vs. group H at the same time; # p <0.05 vs. HF group at the same time.

At the beginning of the protocol, all groups showed similar body weight. There was an increase in body weight throughout the protocol in all groups studied. Moreover, the HF group (at 7 and 15 days of protocol) and the HFTC group (at 15 days of protocol) showed higher body weight than the H group. The HFTC group showed higher body weight at 7 days of protocol when compared to the HF group (Fig 1A). The total caloric intake (chow + fructose) was similar among groups during the protocol (S1 Table).

All groups presented an increase in white adipose tissue weight throughout the protocol. Fructose overload led to an additional increase in white adipose tissue weight after 60 days (HF vs. H). The combined exercise training was able to prevent this additional increase (Fig 1B). The soleus muscle mass was increased in all groups. At 60 days of protocol, the HF group presented lower soleus weight when compared to the H group (Fig 1C). All groups exhibited an increase in the extensor hallucis longus muscle mass throughout at the end of the protocol. The extensor hallucis longus (EHL) muscle mass was higher in the HFTC group when compared to HF at 60 days of protocol (Fig 1D).

**Fig 2:**
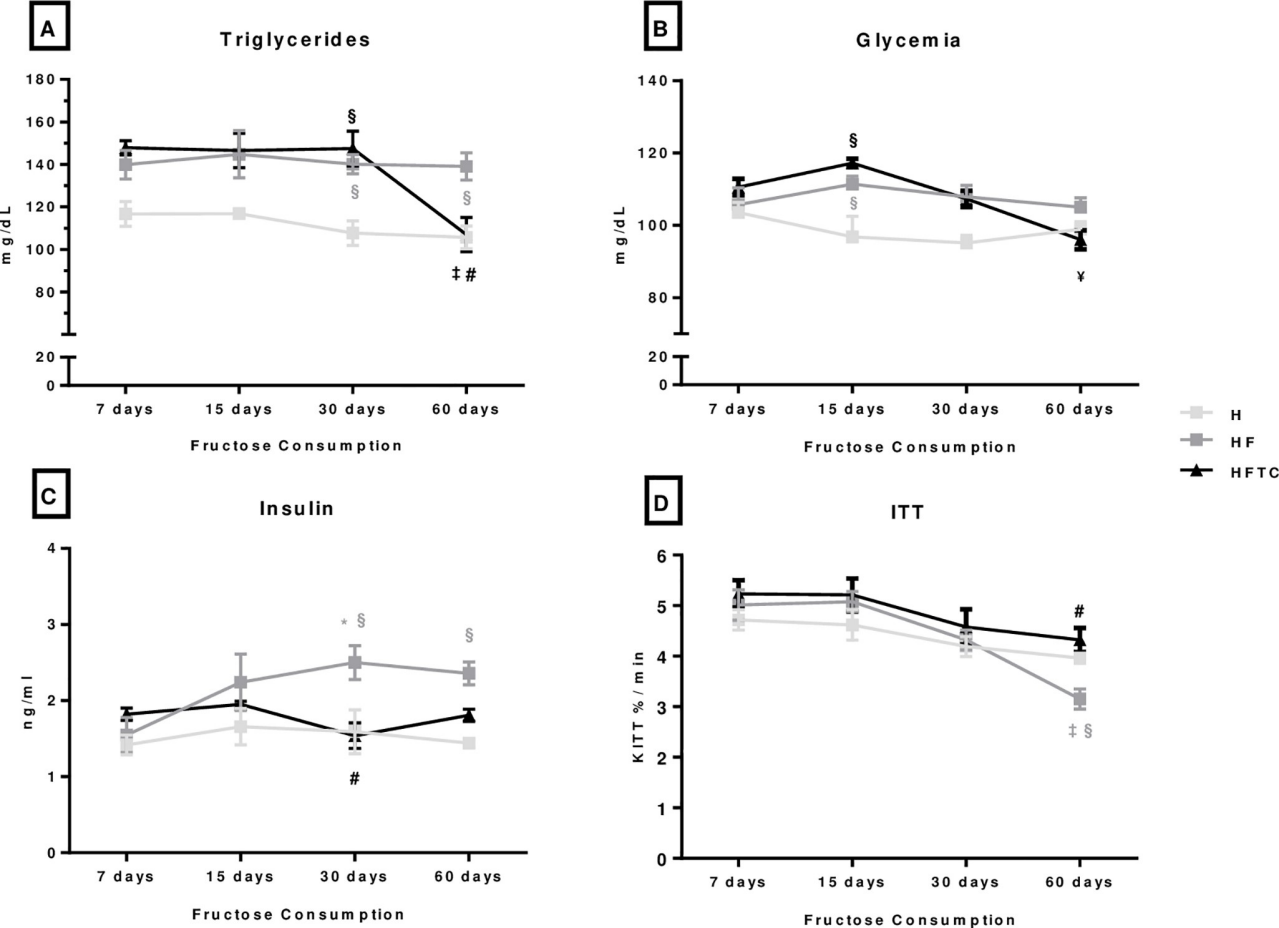
A) triglycerides, B) glycemia, C) insulin and D) ITT of the hypertensive (H), hypertensive + fructose (HF) and hypertensive + fructose + combined physical training (HFT) groups at 7, 15, 30 and 60 days. * p <0.05 vs. 7 days in the same group; ¥ p <0.05 vs.15 days in the same group; § p <0.05 vs. group H at the same time; # p <0.05 vs. HF group at the same time.

Triglyceride levels were higher in the HF and HFTC groups at 30 protocol when compared to the H group. Moreover, HF group showed higher triglyceride levels at 60 days when compared to the H group. However, at the end of the protocol (day 60), the HFTC group presented lower triglyceride levels when compared to the HF group and its initial values (Fig 2A). The HF and HFTC groups presented higher glucose levels on day 15 when compared to the H group. At 60 days of protocol, the HFTC group showed lower glucose levels when compared to its values in day 15 (Fig 2B). Plasma insulin levels were higher in the HF group at 30 days of protocol when compared to its values at 7 days of protocol. Also, they were increased in the HF group when compared to the H group on days 30 and 60 (Fig 2C). At the end of protocol, the HF group presented a reduction in insulin sensitivity when compared to its previous values (days 7, 15 and 30), as well as lower values when compared to the H group on day 60. The HFTC group showed higher insulin sensitivity than the HF group at 60 days of protocol (Fig 2D).

### Hemodynamics measurements

**Fig 3:**
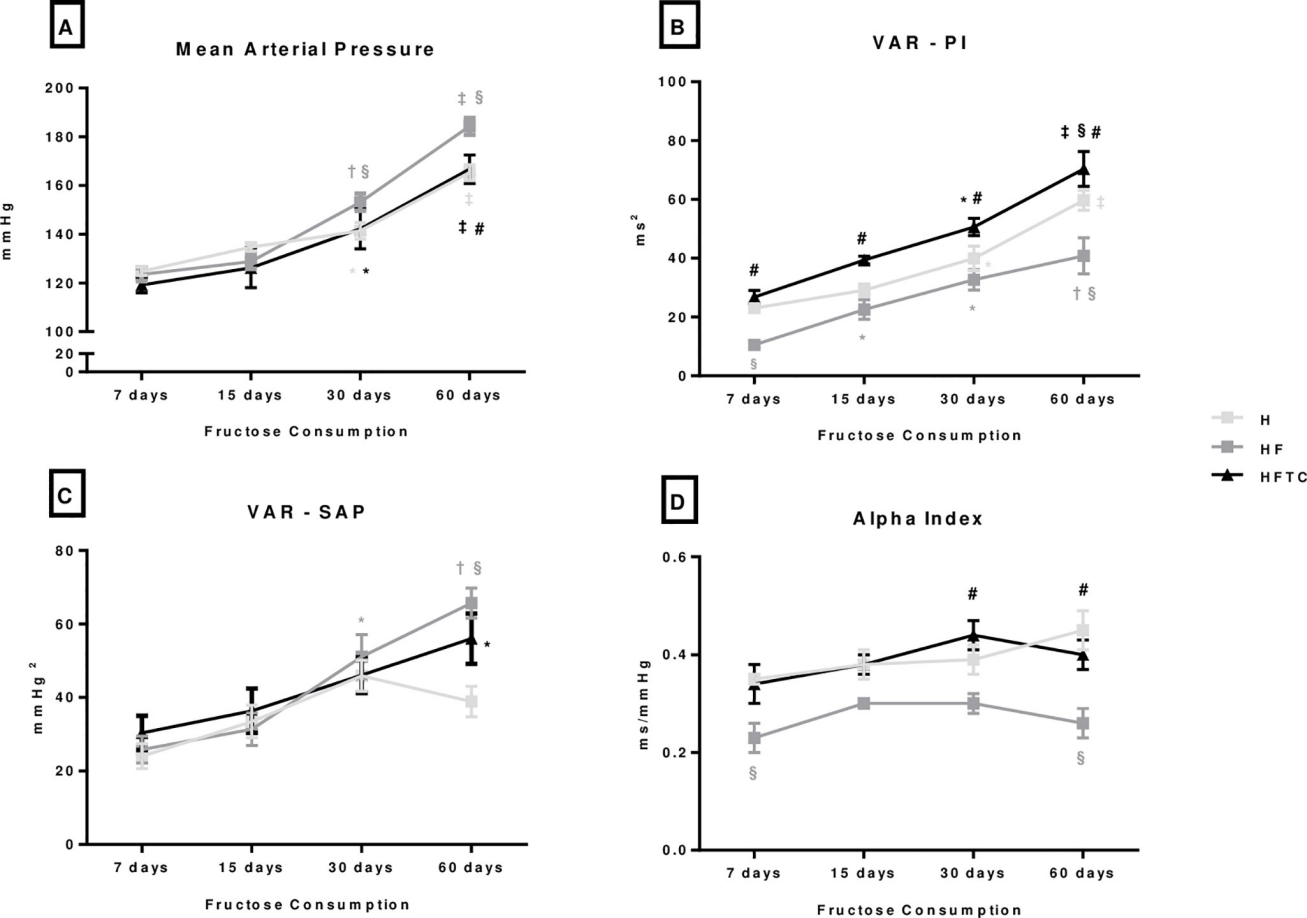
A) mean arterial pressure, B) variance of pulse interval, C) variance of arterial pressure and D) alpha index of the hypertensive (H), hypertensive + fructose (HF) and hypertensive + fructose + combined physical training (HFTC) groups at 7, 15, 30 and 60 days. * p <0.05 vs. 7 days in the same group; † p <0.05 vs. 7 and 15 days in the same group; ‡ p <0.05 vs. 7, 15 and 30 days in the same group; § p <0.05 vs. group H at the same time; # p <0.05 vs. HF group at the same time.

At the end of the protocol, all groups presented an increase in SAP, DAP and MAP when compared to their initial values. SAP, DAP and MAP were increased in the HF group when compared to the H group at 60 days of protocol. The combined exercise capacity prevented this increase in AP induced by the fructose consumption at 60 days of protocol (Fig 3A). Heart rate was lower at 15, 30 and 60 days of protocol in both the H and HF groups, and at 30 and 60 days of protocol in the HFTC group when compared to 7 days (Table 1).

**Table 1:** Arterial pressure and resting heart rate of the Hypertensive (H), hypertensive + fructose (HF) and hypertensive + fructose + combined physical training (HFTC) groups at 7, 15, 30 and 60 days.

Days	7	15	30	60
Variables				
**SAP (mmHg)**				
**H**	144.5±3.1	157.2±2.0	165.7±4.5*	192.0±4.3‡
**HF**	144.6±3.5	150.9±1.7	175.5±3.7†	211.0±6.4‡§
**HFTC**	138.9±4.7	142.8±5.7	165.1±2.1†	199.9±4.4‡
**DAP (mmHg)**				
**H**	103.4±2.9	109.5±1.7	118.7±3.1*	141±4.1‡
**HF**	101.9±3.0	107.4±3.3	132.1±3.9†§	158.9±4.2‡§
**HFTC**	99.5±2.5	110.0±1.1	118.9±3.7*#	137.1±7.1‡#
**HR (bpm)**				
**H**	426.8±13.5	381.0±7.6 *	351.6±7.1*	352.3±9.7*
**HF**	450.3±10.8	389.7±10.9*	371.0±4.2*	376.3±15.7*
**HFTC**	452.6±17.7	406.4±8.1	379.0±8.2*	376.6±5.8*

Values are expressed as means ±SE.

* p <0.05 vs. 7 days in the same group;

^&^ p <0.05 vs. 30 days in the same group;

^†^ p <0.05 vs. 7 and 15 days in the same group;

^‡^ p <0.05 vs. 7, 15 and 30 days in the same group;

^§^ p <0.05 vs. group H at the same time;

^#^ p <0.05 vs. HF group at the same time.

### Cardiac and vascular autonomic modulation

Regarding cardiac modulation, at 7 and 60 days of protocol the HF showed a decreased VAR-PI when compared to the H group (Fig 3B). The HFTC group exhibited higher VAR-PI and RMSSD index in all time periods studied/throughout the study when compared to the HF group. No differences were observed among groups in absolute values of LF and HF band (Table 2).

**Table 2:** Heart rate and systolic arterial pressure variability of the Hypertensive (H), hypertensive + fructose (HF) and hypertensive + fructose + combined physical training (HFTC) groups at 7, 15, 30 and 60 days.

Days	7	15	30	60
Variables				
**RMSSD (ms)**				
**H**	4.54±0.35	4.33±0.22	4.27±0.39	5.78±0.33‡
**HF**	3.17±0.18§	4.69±0.19	4.50±0.34	4.51±0.30§
**HFTC**	4.42±0.23#	5.71±0.28#	6.24±0.28*§#	6.32±0.26*#
**HF abs (ms**^**2**^**)**				
**H**	3.95±0.68	5.12±0.45	5.12±0.46	6.15±0.97
**HF**	3.14±0.47	5.66±0.45	5.39±0.66	4.19±0.52
**HFTC**	3.13±0.64	7.18±0.52*	6.29±0.60	5.40±0.55
**LF abs (ms**^**2**^**)**				
**H**	0.27±0.03	0.60±0.12	0.84±0.12	1.24±0.12†
**HF**	0.48±0.09	1.00±0.07*	1.14±0.15*	1.37±0.17*
**HFTC**	0.26±0.06	0.66±0.12	0.92±0.12	1.19±0.12†
**LF-SAP (mmHg**^**2**^**)**				
**H**	5.14±1.02	5.58±0.94	8.58±2.83	9.71±2.79
**HF**	4.51±0.73	5.94±1.22	9.45±3.16	10.45±2.30
**HFTC**	4.53±0.65	5.75±0.40	8.97±1.88	10.80±2.24

Values are expressed as means ±SE.

* p <0.05 vs. 7 days in the same group;

^&^ p <0.05 vs. 30 days in the same group;

^†^ p <0.05 vs. 7 and 15 days in the same group;

^‡^ p <0.05 vs. 7, 15 and 30 days in the same group;

^§^ p <0.05 vs. group H at the same time;

^#^ p <0.05 vs. HF group at the same time.

At the end of the protocol (day 60), the HF group presented higher VAR-SAP in relation to its values at 7 and 15 days of protocol. Moreover, we observed increased VAR- SAP and decreased alpha index in the HF group when compared to the H group at day 60 (Fig 3C). On the other hand, the HFTC group had a higher alpha index at 30 and 60 days of protocol than the HF group (Fig 3D).

### Plasma nitrite and inflammatory mediators

**Fig 4:**
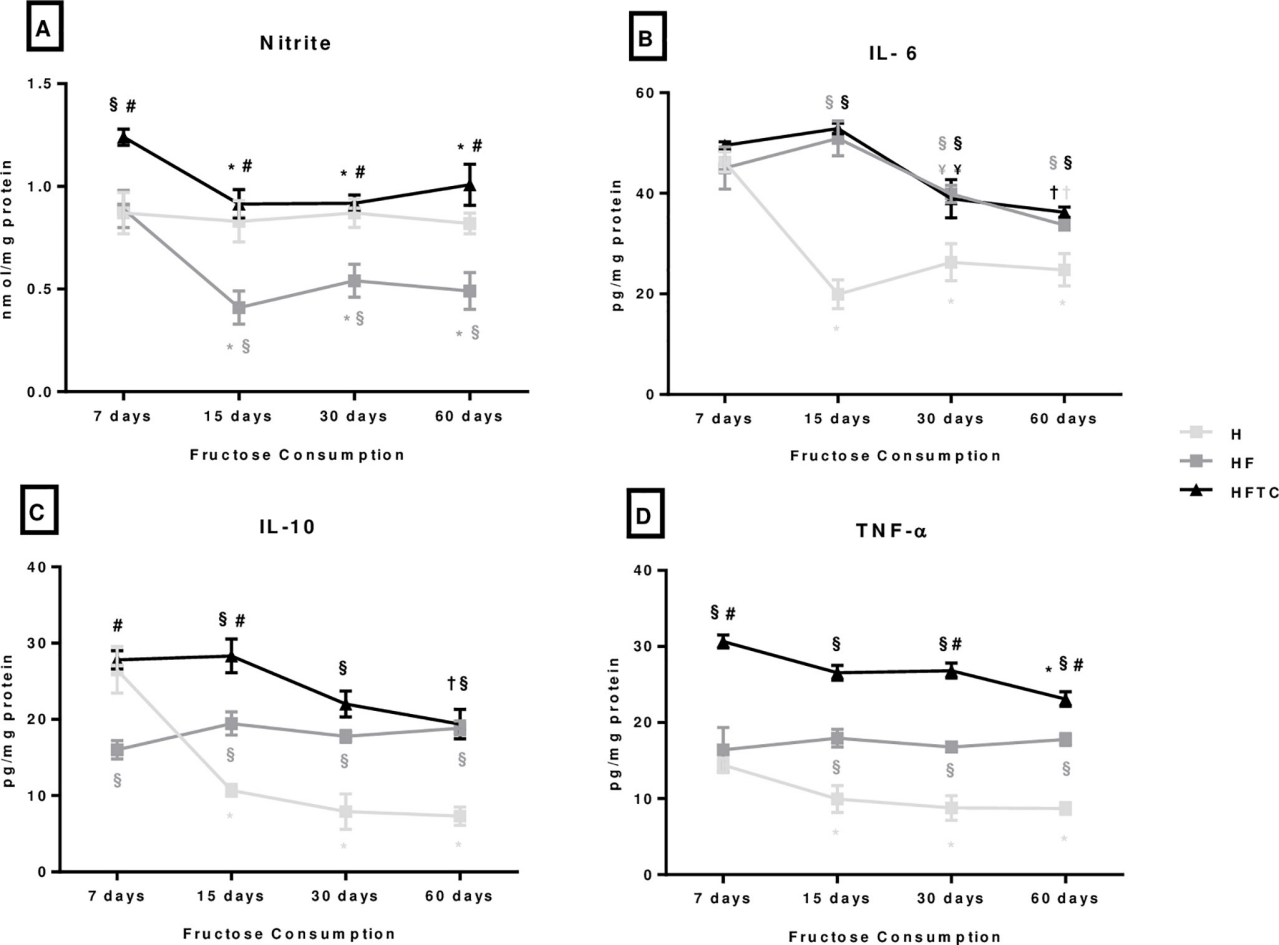
A) nitrite, B) interleukin 6, C) interleukin 10 and D) TNF-α of the hypertensive (H), hypertensive + fructose (HF) and hypertensive + fructose + combined physical training (HFTC) groups at 7, 15, 30 and 60 days. * p <0.05 vs. 7 days in the same group; & p <0.05 vs. 30 days in the same group; † p <0.05 vs. 7 and 15 days in the same group; § p <0.05 vs. group H at the same time; # p <0.05 vs. HF group at the same time.

There was a reduction in plasma nitrite in the HF group at 15, 30 and 60 days of protocol when compared to the H group and to its initial value (day 7). The HFTC group showed higher values of plasma nitrite at 7 days of protocol than the H and HF groups and at 15, 30 and 60 when compared to the HF group (Fig 4A).

The groups undergoing fructose consumption (HF and HFTC groups) presented an increase in IL-6, IL-10 and TNF-alpha at 15, 30 and 60 days of protocol when compared to the H group (Fig 4B, 4C and 4D).

### Oxidative stress profile

**Fig 5:**
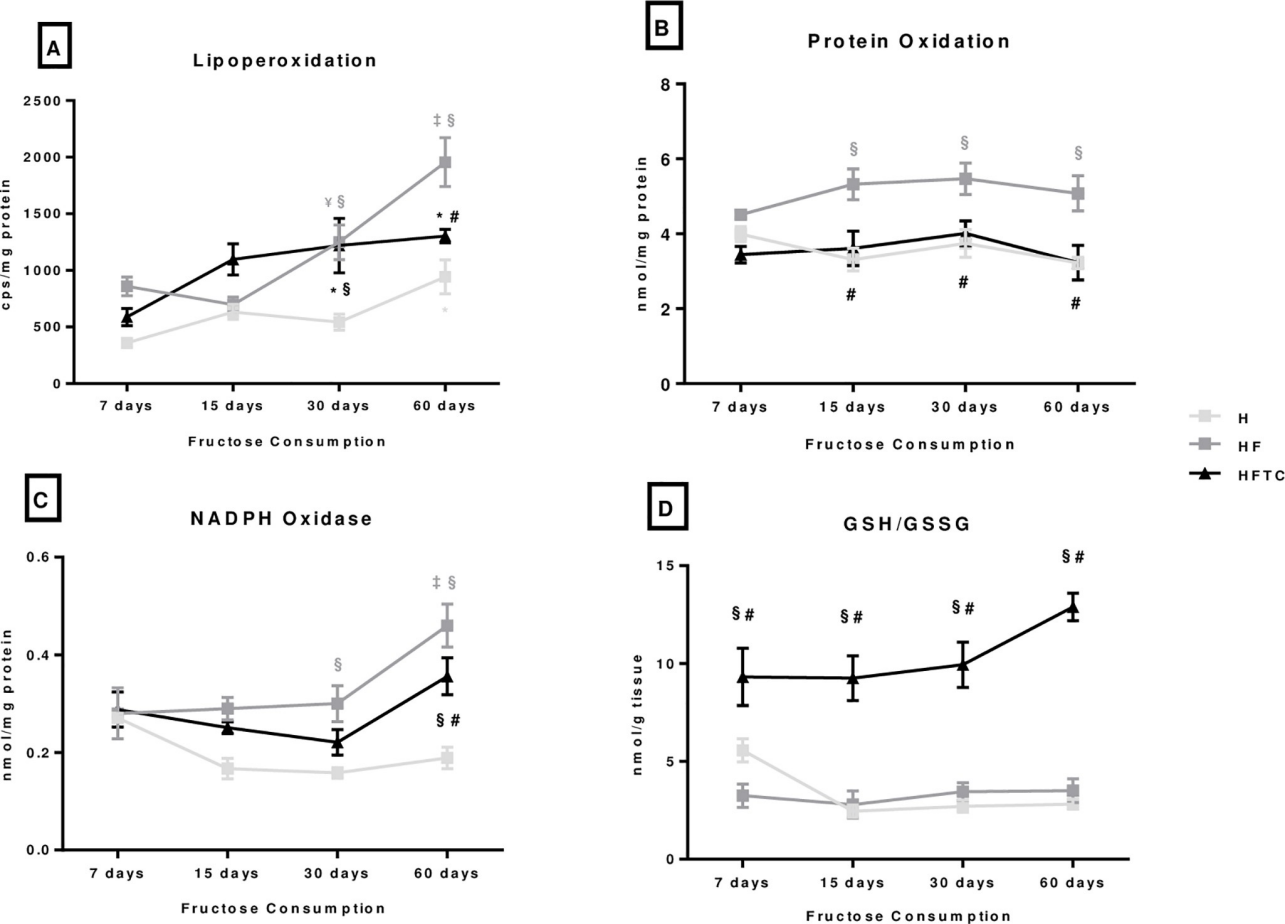
A) lipoperoxidation, B) protein oxidation, C) NADPH oxidase and D) GSH/GSSG of the hypertensive (H), hypertensive + fructose (HF) and hypertensive + fructose + combined physical training (HFTC) groups at 7, 15, 30 and 60 days. * p <0.05 vs. 7 days in the same group; ¥ p <0.05 vs.15 days in the same group; ‡ p <0.05 vs. 7, 15 and 30 days in the same group; § p <0.05 vs. group H at the same time; # p <0.05 vs. HF group at the same time.

There was an increase in LPO in the HF group throughout the protocol (day 60 vs. 7, 15 and 30). Also, at the end of the protocol (day 60), LPO was increased in the HF group when compared to the H group. The combined exercise training was able to prevent the increase promoted by the fructose consumption at the end of protocol (HFTC vs. HF) (Fig 5A). Protein oxidation was increased in the HF group at 15, 30 and 60 days of protocol when compared to the H group. Once more, the combined exercise training was able to prevent the increase promoted by fructose consumption (HFTC vs. HF) (Fig 5B).

At 30 and 60 days of protocol, NADPH oxidase was increased in the HF group when compared to the H group. However, at 60 days the combined exercise training (HFTC group) was able to prevent this increase (HFTC vs. HF) (Fig 5C). The HFTC group showed lower superoxide anion at 7 days of protocol when compared to the H and HF groups, and at 15 days of protocol when compared to the H group (Table 3). At the end of protocol (day 60), hydrogen peroxide was reduced in the HF group when compared to the H group (Table 3).

**Table 3:** Reactive oxygen species and antioxidant enzymes in cardiac tissue of the Hypertensive (H), hypertensive + fructose (HF) and hypertensive + fructose + combined physical training (HFTC) groups at 7, 15, 30 and 60 days.

Days	7	15	30	60
Variables				
**Superoxide Anion** (nmol/mg protein)				
**H**	9.58± 0.71	7.84± 0.58	8.01±1.01	5.29± 0.62*
**HF**	8.44± 1.01	7.04± 0.90	7.23±0.54	5.12± 0.75*
**HFTC**	4.25± 0.47#§	4.14± 0.56§	5.29±0.62	3.81± 0.29
**Hydrogen peroxide** (μM)				
**H**	4.83±2.33	3.03±0.84	1.87±0.54	2.06±0.57
**HF**	5.35± 1.26	4.63±1.13	4.35±0.48	6.34±0.89§
**HFTC**	5.80±0.71	4.94±0.89	5.46±0.61	4.90±0.61
**CAT** (nmol/mg protein)				
**H**	0.70± 0.03	0.66±0.05	0.66±0.05	0.67± 0.05
**HF**	0.60± 0.03	0.64± 0.06	0.50±0.04	0.46± 0.06
**HFTC**	0.74± 0.04	0.71± 0.09	0.65±0.03	0.68± 0.04
**GPx** (μmol/min/mg protein)				
**H**	0.04± 0.007	0.04±0.006	0.04±0.008	0.05± 0.009
**HF**	0.04± 0.011	0.04± 0.009	0.05±0.011	0.05± 0.009
**HFTC**	0.03± 0.002	0.03± 0.002	0.04±0.006	0.07± 0.005†
**SOD** (USOD/mg protein)				
**H**	16.18±1.25	17.96±0.89	16.34±1.08	16.61±0.95
**HF**	16.43±1.23	16.03±0.63	18.03±0.56	15.67±0.79
**HFTC**	14.83±0.79	14.01±0.98	16.02±0.91	15.02±1.04

Values are expressed as means ±SE.

^†^ p <0.05 vs. 7 and 15 days in the same group;

^#^ p <0.05 vs. HF group at the same time.

No differences were observed among the groups and periods regarding CAT and SOD. GPx was higher in the HFTC group at 60 days of protocol when compared to 7 days of protocol in the same group.

GSH/GSSG ratio was higher 7, 15, 30 and 60 days of protocol for the HFTC group when compared to the H and HF groups (Fig 5D).

## Discussion

In this study we found early cardiovascular autonomic dysfunction (at day 7) followed by impairment in inflammatory and oxidative stress markers (15–60 days), resulting in late cardiometabolic changes (30–60 days) induced by fructose consumption in genetically predisposed rats to hypertension. However, the more relevant and new finding of our study lies in that combined exercise training initiated early in life may prevent premature HRV and baroreflex impairments, and may promote positive changes in inflammatory and oxidative stress profiles in cardiac tissue, resulting in prevention of AP, triglyceride and insulin resistance and the negative changes induced by fructose overload in SHR.

In fact, The HF group presented a reduction in alpha index at 7 and 60 days when compared to the H group. Importantly, combined exercise training was able to increase alpha index, which was reduced in the HF group at 30 and 60 days of protocol. We have previously demonstrated in that aerobic or resistance exercise training improved baroreflex sensitivity for tachycardic responses in adult SHR with ovarian hormone deprivation; however, only aerobic exercise training was effective to improve baroreflex mediated-bradycardic response and alpha index [8]. Additionally, using an adult male SHR model, Masson et al. [9] have indicated that aerobic exercise training restored baroreflex sensitivity in the first 2 weeks of the training protocol, regardless of baseline blood pressure levels, indicating that normalization of baroreflex control is the first adaptive response of the cardiovascular system to exercise training and precedes the occurrence of bradycardia resting and reduced blood pressure.

In the present study, a decrease in VAR-PI, which is associated with vagal modulation in the heart, was observed at 7 and 60 days in the HF group when compared to the H group. Combined exercise training was able to prevent this reduction. Moreover, the HFTC group showed increased VAR-PI at 7, 15 and 30 days of protocol when compared to the HF group in the same periods. Findings from our group have shown that 19 weeks of fructose overload in females SHR associated with ovarian hormone deprivation induced a reduction in VAR-PI [22]. In addition, a reduction in RMSSD (cardiac parasympathetic modulation representative) was observed in the HF group at 7 and 60 days of protocol when compared to the control group in the same periods. On the other hand, the HFTC presented an increase in the BRS index when compared to the HF in the same time periods and when compared to the H group at 15 and 30 days. It is worth mentioning that parasympathetic dysfunction was associated with insulin resistance in Wistar rats undergoing fructose consumption for 8 weeks [13].

Regarding cardiovascular parameters, AP was increased in all groups at 30 and 60 days of protocol when compared to their initial values at 7 and 15 days. Fazan et al. [23] have observed that SHR begin to develop hypertension at 5 weeks of age. In the present study, fructose consumption induced an additional increase in AP at 60 days of protocol when compared to the control group in the same period. In this aspect, it should be mentioned that a systematic review, involving more than 400.000 research subjects, showed that sugary beverage intakes were significantly associated with higher AP and increased incidence of hypertension [24]. Increased AP in animal models with fructose overload has been demonstrated in mice [3], normotensive male rats [25] and hypertensive female rats [6].

It is important to remind that pharmacological approaches provide the primary basis for treatment of high AP. A large number of clinical trials have shown that antihypertensive pharmacotherapy not only reduces BP, but also reduces the risk of CVD, cerebrovascular events, and death. A recent meta-analysis involving 391 randomized controlled trials assessing exercise and medications effects on SAP, demonstrated that in populations with hypertension, different types of exercise interventions (aerobic, resistance, combination) appear to be equally effective (SAP reduction <10 mmHg) as most antihypertensive medications (ACE inhibitors, beta-blockers, diuretics, ARB, CCB) in monotherapy [26]. In this aspect, in the present paper we demonstrated that combined exercise training was effective in reducing the additional DAP and MAP increase observed in genetic predisposed hypertensive rats submitted to chronic fructose consumption, suggesting a positive role of this approach to attenuate hypertension development.

One mechanism which may account for fructose-induced AP increase would be that excess dietary fructose leads to chronic stimulation of the sympathetic nervous system, primarily as a result of increased insulin levels. In turn, overactivation of the sympathetic nervous system is believed to exacerbate insulin resistance, thereby setting up a positive feedback loop [27]. In fact, insulin resistance and vascular sympathetic modulation were higher in the HF group in this study. VAR-SAP (which represents the vascular sympathetic modulation), after 60 days of fructose consumption, was higher in the HF group than the H group and it was accompanied by impaired spontaneous baroreflex sensitivity (alpha index) and increased AP in this period. However, it should be stressed that the first alterations induced by fructose overload in SHR in the present study was the impairment of both baroreflex and VAR-PI, suggesting that this may trigger other neuroimmune changes [5].

Although the trained group showed no reduction in body weight when compared to the fructose group (HF) at the end of the protocol, a reduction in retroperitoneal adipose tissue was observed in the trained animals in the same period (60 days). A similar reduction was also observed by Farah et al. [25] in animals undergoing aerobic exercise training (AT), after 8 weeks of protocol. Stanhope et al. [28] have shown that fructose-sweetened beverages (but not glucose-sweetened beverages) stimulated intra-abdominal lipid deposition and hepatic lipid production, while cholesterol metabolism was negatively impacted, and insulin sensitivity was reduced, suggesting that fructose consumption may specifically induce/cause lipid deposition in visceral adipose tissues.

In our study, fructose consumption induced an increase in triglycerides at 30 and 60 days of protocol when compared to the H group. Triglycerides in plasma are derived from fats eaten in foods or from other energy sources. An excess of triglycerides in plasma is positively and independently associated with cardiovascular disease [29]. Several studies have demonstrated an increase in triglycerides after a protocol of fructose overload, both in experimental models [13,22,25] and in humans [28,30]. On the other hand, it was demonstrated that the late cardiometabolic changes promoted by combined exercise training was effective in reducing triglyceride values at 60 days of protocol when compared to the sedentary group (HF). In a previous study, our group has also observed a reduction in triglyceride values after a protocol of combined exercise training in a model of menopause and metabolic syndrome [31]

The exposure of the liver to such large quantities of fructose leads to rapid stimulation of lipogenesis and triglycerides accumulation, which in turn contributes to reduced insulin sensitivity and hepatic insulin resistance/glucose intolerance, leading to an increase of circulating insulin [32]. In the present study, insulin was higher at days 30 and 60 in the fructose group (HF) when compared to the control group (H). After 60 days of fructose overload, the rats had increased in insulin when compared to both the group receiving fructose for only15 days and to the control group [3].

Regarding insulin sensitivity, the HF group presented a reduction at 60 days vs. 7, 15 and 30 days of protocol. Additionally, the HF group had higher insulin resistance when compared to the H group at 60 days of protocol. Combined exercise training was able to improve insulin sensitivity at the end of the protocol. Conti et al. [22] have demonstrated that fructose consumption promotes a reduction in insulin sensitivity in a model of metabolic syndrome and menopause. Improved insulin sensitivity was [+also] demonstrated after a protocol of aerobic exercise training [7] or combined exercise training [31] in a model of metabolic syndrome.

It should be emphasized that vagal activity can modulate inflammatory response and oxidative stress in some pathophysiological situations [33,34]. In this sense, we hypothesized that the autonomic improvement induced by combined aerobic training in fructose SHR group may have modulated inflammatory responses in our study. Our findings show that the HF group presented an increase in TNF-α at the end of the protocol when compared to 7 days, and at 15, 30 and 60 days of protocol when compared to the H group. Indeed, fructose overload may lead to an increase in TNF-α in adipose tissue in menopaused SHR [22], increased vascular TNF-α in male SHR [35], and increased TNF liver expression of normotensive mice [36]. Moreover, fructose overload induced an increase in IL- 6 and TNF-α cytokines. Additionally, combined exercise training induced an increase in IL-10, a cytokine related to the anti-inflammatory profile.

The excessive consumption of fructose causes metabolic dysfunctions, due to inflammation and oxidative stress, which are related to increased cardiovascular risk [37]. In the present study, the consumption of fructose, despite unchanged antioxidant enzyme activity, induced an increase in lipoperoxidation values in the heart tissue only at 30 and 60 days of protocol in relation to the H group. However, combined exercise training was able to prevent this increase at 60 days of protocol when compared to both H and HF groups. Conti et al. [22] have also observed in female SHR, that fructose overload led to an increased cardiac lipoperoxidation detected by 2 different techniques, TBARS and chemiluminescence (QL). QL was decreased in the HFTC group in the present study. There was a higher oxidation of proteins in the HF group at 15, 30 and 60 days of protocol when compared to the H group. There was reduction in protein oxidation in the HFTC group when compared to the HF group in the same periods, indicating lower protein injury. Farah et al. [25] have demonstrated that plasma protein oxidation was increased in normotensive rats, but aerobic exercise training was able to improve it and reduce protein oxidation in cardiac tissue.

It should be noted that the toxicity of fructose is directly related to the time of exposure to this sugar. In animals consuming fructose (30%) for 24 weeks an increase in oxidative stress was observed, whereas the group that stopped consuming fructose improved the parameters of oxidative stress [38]. Although fructose is metabolized in the liver, this does not exclude its deleterious effects on other organs such as the heart, kidneys and brain. Morphological and functional changes in the kidneys were observed after consumption of fructose resulting in hyperfiltration, hyperplasia of mesangial cells in glomerulus [39] glomerular hypertension, cortical vasoconstriction, and arteriolopathy of preglomerular vessels and kidney hypertrophy [40]. These changes were probably associated with increase in protein and lipid oxidation (oxidative stress damage markers) in renal tissue in fructose-fed animals [31]. Moreover, the increase in reactive oxygen species, lipoperoxidation and NADPH oxidase, accompanied by a suppression of superoxide dismutase activity, was observed in the brain of rats (RVLM) [41]. In the presence of NADPH oxidase, an electron of NADPH is transferred to an oxygen molecule, generating the final product of this reaction, the superoxide anion. The superoxide anion inactivates NO for peroxynitrite production and spontaneously or isolated of superoxide dismutase reacts with hydrogen peroxide, which, in the present study, was increased at 60 days of protocol in the HF group when compared to the H group. Giris et al. [42] have observed an increase in hepatic non-tissue hydrogen peroxide from rats undergoing 8 weeks of fructose consumption. In the present study, combined exercise training attenuated the increase in NADPH oxidase activity induced by fructose overload at 60 days, and was able to reduce superoxide anion at 7 and 15 days of protocol.

We also evaluated the redox balance by the GSH/GSSG ratio, a key assessment tool that demonstrates a better relationship between pro and antioxidant. The HFTC group presented higher GSH/GSSG ratio at 60 days when compared to its values at 7, 15 and 30 days of protocol. Some studies have also found an increase in the GSH/GSSG ratio after an exercise training protocol [25,31]. Kihlström [43] have demonstrated a protective effect on cardiac oxidative stress in rats undergoing a protocol of swimming, along with a reduction in GSSG and an increase in GSH. Thus, the increased GSH /GSSG ratio, the attenuation of NADPH oxidase and the reduction of protein oxidation and lipoperoxidation observed in the HFTC group indicates a better oxidative profile when compared to the sedentary group.

In conclusion, our findings demonstrated that combined exercise training performed simultaneously with exacerbated fructose consumption during a lifespan prevents early cardiovascular autonomic dysfunction, probably by triggering positive changes in inflammation and oxidative stress, resulting in better cardiometabolic profile in rats genetically predisposed to hypertension.

## Supporting information

S1 Table Drink, chow and calories of the Hypertensive (H), hypertensive + fructose (HF) and hypertensive + fructose + combined physical training (HFTC) groups at 7, 15, 30 and 60 days. (PDF)

S1 Dataset (PDF)
