# Correction: Impact of combined exercise training on the development of cardiometabolic and neuroimmune complications induced by fructose consumption in hypertensive rats

**DOI:** 10.1371/journal.pone.0235983

**Published:** 2020-07-06

**Authors:** Danielle da Silva Dias, Nathalia Bernardes, Filipe Fernandes Stoyell-Conti, Camila Paixão dos Santos, Amanda Aparecida de Araujo, Susana Llesuy, Maria Cláudia Irigoyen, Kátia De Angelis

In the Funding statement, the grant numbers from the funder Fundação de Amparo à Pesquisa do Estado de São Paulo (FAPESP) are listed incorrectly. The correct grant numbers are: 2020/07904-6; 2018/17183-4; 2015/11223-6.
