# Supplementary material for: Impact of combined exercise training on the development of cardiometabolic and neuroimmune complications induced by fructose consumption in hypertensive rats
Source: PLoS One. 2020 Jun 10;15(6):e0233785. doi: 10.1371/journal.pone.0233785 (PMC7286703; doi:10.1371/journal.pone.0233785)
Supplement: S1 Table — (PDF) [file pone.0233785.s001.pdf]

Table S1. Drink, chow and calories of the hypertensive (H), hypertensive + fructose (HF) and hypertensive + fructose + combined physical training (HFTC) groups at 7, 15, 30 and 60 days.

Day / Variables	7	15	30	60
**Drink (ml/day/rat)**				
**H**	14.9 ±2.3	25.8±3.9	25.9±1.2	29.9±2.4*
**HF**	19.6±0.9	25.0±2.7	32.7±3.9	38.2±2.4*
**HFTC**	21.9±3.6	25.6±4.6	32.1±4.2	39.3±2.0*¥
**Chow (g/day/rat)**				
**H**	9.7±1.7	12.4±0.4	19.6±1.0*¥	20.7±1.2*¥
**HF**	9.6±0.6	9.9±0.9	13.0±2.3§	13.5±1.9§
**HFTC**	11.5±1.6	13.5±1.3	13.7±1.0§	13.8±0.8§
**Calories (kcal/day/rat)**				
**H**	28.1±4.9	35.8±1.2	56.7±3.0*	59.9±3.4*¥
**HF**	35.6±2.2	38.9±3.8	51.1±8.1	54.3±6.2
**HFTC**	42.1±6.2	49.2±5.5	52.5±4.7	54.9±3.3

Values are expressed as means ±SE. * p <0.05 vs. 7 days in the same group. ¥ p <0.05 vs. 15 days in the same group. § p <0.05 vs. group H at the same time.
