# Supplementary material for: Impact of combined exercise training on the development of cardiometabolic and neuroimmune complications induced by fructose consumption in hypertensive rats
Source: PLoS One. 2020 Jun 10;15(6):e0233785. doi: 10.1371/journal.pone.0233785 (PMC7286703; doi:10.1371/journal.pone.0233785)
Supplement: S1 Dataset — (PDF) [file pone.0233785.s002.pdf]

Body weight, white adipose tissue weight, soleus mass and extensor hallucis longus (EHL) mass of the hypertensive (H), hypertensive + fructose (HF) and hypertensive + fructose + combined physical training (HFTC) groups at 7, 15, 30 and 60 days.

Days / Variables	7	15	30	60
**Body weight** (g)				
**H**	71.54±4.47	94.09±4.31	176.11±1.62	259.64±5.61
**HF**	95.71±2.96	115.45±3.60	172.50±8.21	258.92±4.82
**HFTC**	77.57±5.94	117.68±6.80	167.00±9.60	264.22±5.02
**White adipose tissue** (g)				
**H**	0.06±0.02	0.12±0.04	0.65±0.08	1.61±0.11
**HF**	0.11±0.02	0.25±0.02	0.64±0.09	1.91±0.10
**HFTC**	0.07±0.01	0.27±0.03	0.59±0.13	1.42±0.13
**Soleus mass (g)**				
**H**	0.033±0.002	0.050±0.003	0.081±0.002	0.111±0.003
**HF**	0.033±0.003	0.050±0.002	0.073±0.004	0.094±0.006
**HFTC**	0.031±0.002	0.047±0.007	0.083±0.007	0.104±0.004
**EHL mass** (g)				
**H**	0.033±0.002	0.051±0.004	0.084±0.002	0.114±0.002
**HF**	0.035±0.003	0.052±0.003	0.080±0.002	0.108±0.002
**HFTC**	0.032±0.002	0.058±0.002	0.080±0.005	0.118±0.004

Triglycerides, glycemia, insulin and ITT of the hypertensive (H), hypertensive + fructose (HF) and hypertensive + fructose + combined physical training (HFT) groups at 7, 15, 30 and 60 days.

Days / Variables	7	15	30	60
**Triglycerides** (mg/dL)				
**H**	116±5.8	116±2.0	107±5.7	105±5.3
**HF**	139.8±6.7	144±11.1	140±4.5	139±6.4
**HFTC**	147±3.3	146±8.0	147±8.1	107±8.0
**Glycemia** (mg/dL)				
**H**	103±2.8	96.±5.7	95±2.1	99±2.1
**HF**	105±4.6	111±2.2	107±3.1	105±2.6
**HFTC**	110±2.3	117±1.1	107±2.2	96±2.6
**Insulin** (mg/ml)				
**H**	1.42±0.13	1.66±0.24	1.59±0.29	1.44±0.06
**HF**	1.55±0.23	2.24±0.37	2.50±0.22	2.36±0.15
**HFTC**	1.82±0.08	1.95±0.04	1.54±0.17	1.81±0.08
**ITT** (KITT%/min)				
**H**	4.72±0.2	4.62±0.3	4.19±.2	4.0±0.1
**HF**	5.0±0.3	5.1±0.2	4.32±0.2	3.15±0.2
**HFTC**	5.23±0.2	5.21±0.3	4.58±0.3	4.32±0.2

Mean arterial pressure, variance of pulse interval (VAR-PI), variance of arterial pressure (VAR-AP) and alpha index of the hypertensive (H), hypertensive + fructose (HF) and hypertensive + fructose + combined physical training (HFTC) groups at 7, 15, 30 and 60 days.

Days / Variables	7	15	30	60
**Mean arterial pressure** (mmHg)				
**H**	124±1.7	134±1.7	141±3.4	165±3.2
**HF**	123±2.7	128±2.9	153±3.5	184±3.4
**HFTC**	119±3.2	126±8.1	142±8.2	166±5.8
**VAR-PI** (ms^2)				
**H**	23±1.4	29±2.1	39±4.1	59±3.4
**HF**	10±1.2	22±3.3	32±3.4	40±4.1
**HFTC**	26±2.2	39±1.3	50±2.9	70±5.9
**VAR-AP** (ms^2)				
**H**	24±3.4	33±4.4	45±4.2	39±4.1
**HF**	25±3.7	31±4.4	51±6.0	65±4.1
**HFTC**	30±4.6	36±6.0	46±4.8	56±6.8
**Alpha index** (ms/mmHg)				
**H**	0.35±0.01	0.38±0.03	0.39±0.3	0.45±0.4
**HF**	0.23±0.03	0.30±0.01	0.30±0.02	0.26±0.03
**HFTC**	0.34±0.04	0.38±0.02	0.44±0.03	0.40±0.03

Nitrite, interleukin 6 (IL-6), interleukin 10 (IL-10) and TNF-α of the hypertensive (H), hypertensive + fructose (HF) and hypertensive + fructose + combined physical training (HFTC) groups at 7, 15, 30 and 60 days.

**Variables** \ **Days**	**7**	**15**	**30**	**60**
**Nitrite** (nmol/mg protein)				
**H**	0.87±0.1	0.83±0.1	0.87±0.07	0.82±0.05
**HF**	0.89±0.09	0.41±0.08	0.54±0.08	0.49±0.09
**HFTC**	1.24±0.04	0.91±0.07	0.91±0.04	1.01±0.1
**IL-6** (pg/mg protein)				
**H**	46±2	20±3	26±3	24±3
**HF**	45±4	51±3	39±1	33±1
**HFTC**	49±1	53±1	39±4	36±1
**IL-10** (pg/mg protein)				
**H**	26±3	10±1	8±2	7±1
**HF**	16±1	19±1	17±1	19±1
**HFTC**	28±1	28±2	22±1	19±2
**TNF- α** (pg/mg protein)				
**H**	14.4±0.9	9.9±1.7	8.7±1.6	8.7±0.7
**HF**	16.4±2.9	17.9±1.1	16.7±0.6	17.6±0.7
**HFTC**	30.6±0.8	26.5±0.9	26.9±0.9	23.1±0.9

Lipoperoxidation, protein oxidation, NADPH oxidase and GSH/GSSG of the hypertensive (H), hypertensive + fructose (HF) and hypertensive + fructose + combined physical training (HFTC) groups at 7, 15, 30 and 60 days

Variables \ Days	7	15	30	60
**Lipoperoxidation** (cps/mg protein)				
**H**	359±38	633±65	542±70	942±150
**HF**	858±81	696±65	1248±152	1956±214
**HFTC**	587±75	1096±136	1218±240	1302±58
**Protein Oxidation** (nmol/mg protein)				
**H**	3.9±0.2	3.3±0.3	3.7±03	3.2±0.1
**HF**	4.5±0.1	5.3±0.4	5.4±0.4	5.1±0.4
**HFTC**	3.4±0.2	3.6±0.4	4.0±0.3	3.2±0.4
**NADPH Oxidase** (nmol/mg protein)				
**H**	0.27±0.01	0.16±0.02	0.16±0.01	0.19±0.02
**HF**	0.28±0.05	0.29±0.02	0.30±0.04	0.46±0.04
**HFTC**	0.29±0.036	0.25±0.01	0.22±0.02	0.35±0.04
**GSH/GSSG** (nmol/g protein)				
**H**	5.57±0.59	2.46±0.31	2.71±0.31	2.82±0.25
**HF**	3.25±0.59	2.80±0.69	3.46±0.45	3.51±0.60
**HFTC**	9.32±1.46	9.25±1.115	9.94±1.16	12.90±0.70
